# Supplementary material for: Carcinoma-Associated Fibroblasts Accelerate Growth and Invasiveness of Breast Cancer Cells in 3D Long-Term Breast Cancer Models
Source: Cancers (Basel). 2024 Nov 15;16(22):3840. doi: 10.3390/cancers16223840 (PMC11593312; doi:10.3390/cancers16223840)
Supplement: Supplementary file 1 [file cancers-16-03840-s001.zip › cancers-3285789-supplementary.pdf]

cancers

MDPI

Supplementary Materials

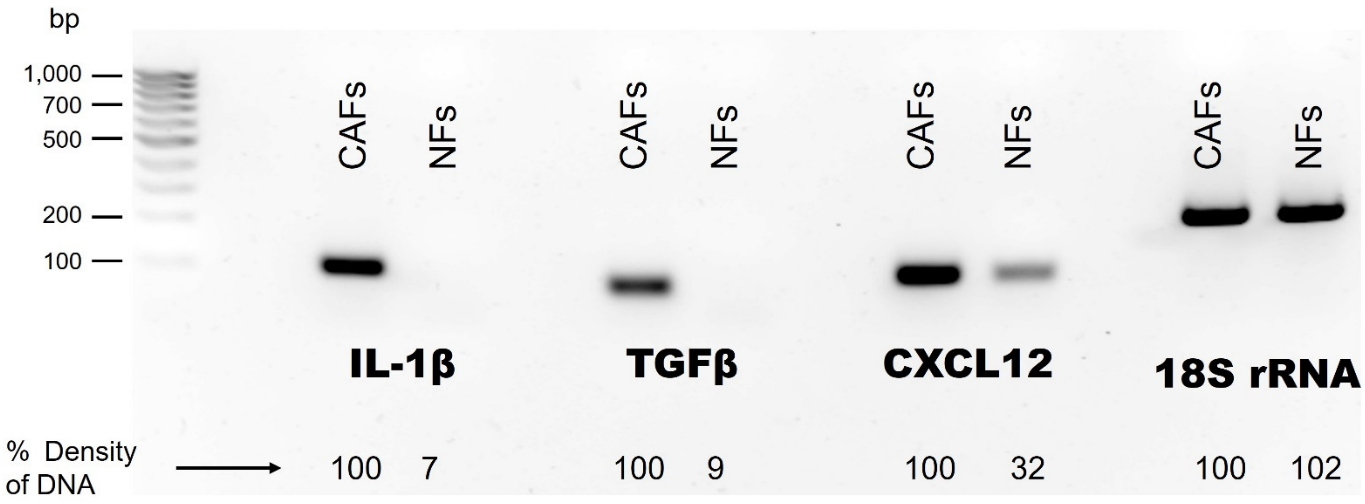

Figure S1. RT- PCR analysis of differentially expressed markers (Interleukin-1 beta, transforming growth factor beta and CXCL12) used to characterize fibroblasts cell lines. CAFs overexpress each of these markers. The internal control used was 18S ribosomal RNA.

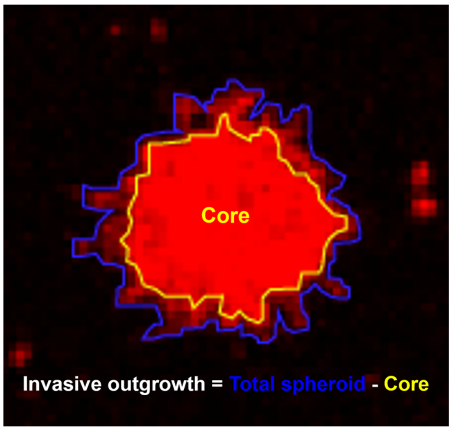

Figure S2. Quantitation of area of cores and invasive outgrowths from BCa spheroids using ImageJ.

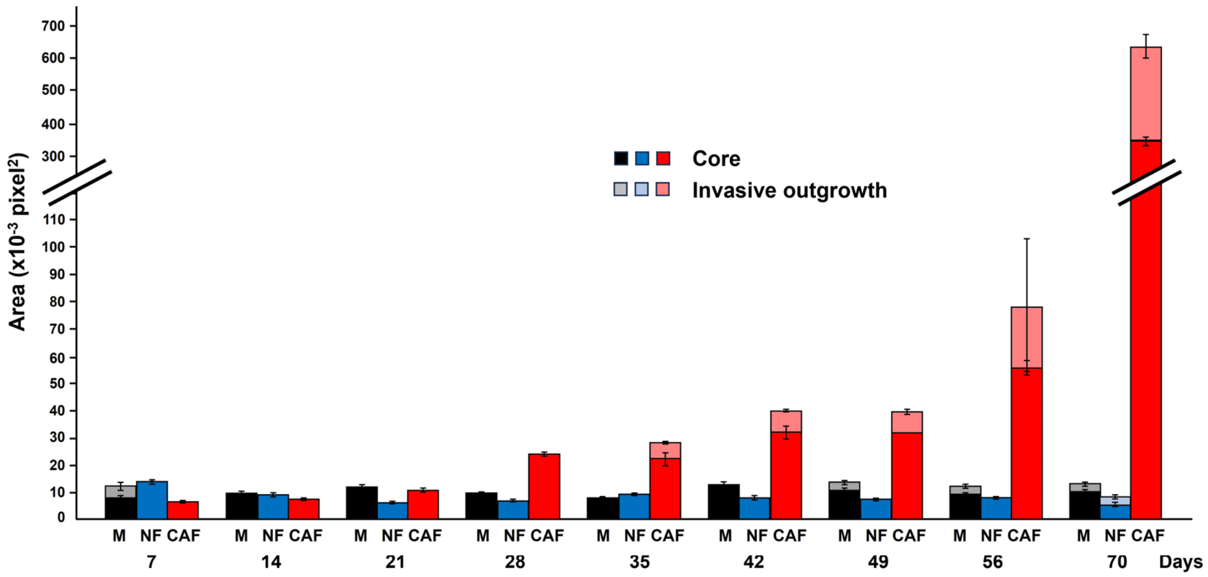

Figure S3. Time-course change of areas for cores and invasive outgrowth from 231 spheroids in monocultures of 231 (M), and parallel cocultures of 231 and NFs (NF) or CAFs (CAF).

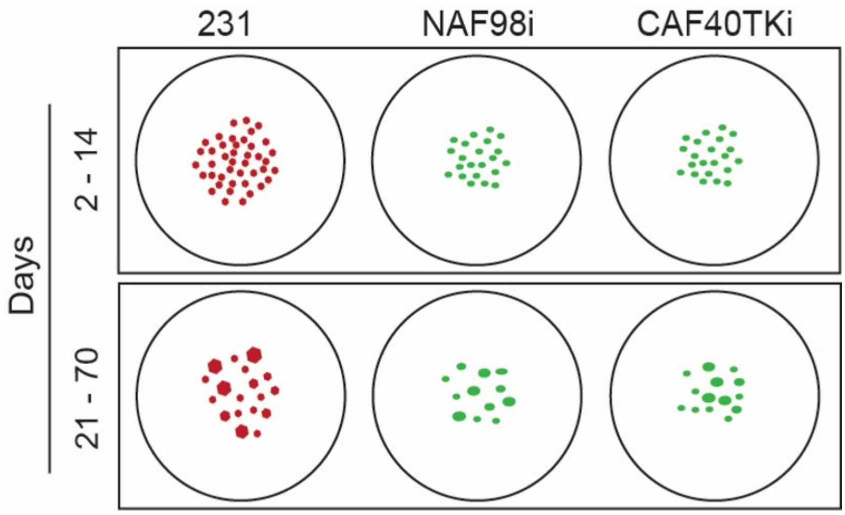

Figure S4. Schematics demonstrating observed changes in spatial distribution and cell cluster size in monocultures of 231, NFs, and CAFs at indicated times.
